# Application of the SYNTAX score in interventional cardiology: A systematic review and meta-analysis

**DOI:** 10.1097/MD.0000000000007410

**Published:** 2017-07-14

**Authors:** Pravesh Kumar Bundhun, Yashna Sookharee, Anita Bholee, Feng Huang

**Affiliations:** a Institute of Cardiovascular Diseases, the First Affiliated Hospital of Guangxi Medical University, Nanning, Guangxi; b Tongji Hospital of Huazhong University of Science and Technology, Wuhan, Hubei, P.R. China.

**Keywords:** coronary artery disease, interventional cardiology, percutaneous coronary intervention, SYNTAX score

## Abstract

**Background:**

Should the SYNTAX score be integrated in Interventional Cardiology? Should it really be considered as a vital decision-making tool in percutaneous coronary intervention (PCI)? To confirm the importance of this score, we aimed to systematically compare the postinterventional adverse cardiovascular outcomes which were observed in patients who were allotted a low versus a high SYNTAX score.

**Methods:**

Randomized controlled trials and observational studies which were published from January 2007 to January 2017 were identified from MEDLINE, EMBASE, and the Cochrane databases using the searched terms ‘SYNTAX score and percutaneous coronary intervention.’ Adverse cardiovascular outcomes were considered as the major endpoints. Risk ratios (RRs) with 95% confidence intervals (CIs) were used as the statistical parameters, and the main analysis was carried out by the RevMan 5.3 software.

**Results:**

Sixteen studies with a total number of 19,751 participants (8589 participants with a low versus 11,162 participants with a high SYNTAX score) were included. Current results showed mortality to be significantly higher with a higher SYNTAX score (RR 2.09, 95% CI 1.78–2.46, *P* = .00001). Cardiac death also significantly favored a low SYNTAX score (RR 2.08, 95% CI 1.66–2.61, *P* = .00001. Similarly, myocardial infarction, major adverse cardiac events, repeated revascularization, and stent thrombosis were significantly higher following a high SYNTAX score (RR 1.71, 95% CI 1.45–2.03, *P* = .00001; RR 2.03, 95% CI 1.81–2.26, *P* = .00001; RR 1.96, 95% CI 1.69–2.28, *P* = .00001; and RR 3.16, 95% CI 2.17–4.59, *P* = .00001, respectively). Even when patients with ST-segment elevation myocardial infarction were separately analyzed, a low SYNTAX score was still significantly associated with lower adverse outcomes.

**Conclusions:**

This analysis is a confirmatory piece of evidence to show that the application of the SYNTAX score in Interventional Cardiology is apparently relevant. The use of this scoring system to grade patients with coronary artery disease and to further guide for revascularization should be encouraged.

## 1 Introduction

What is Interventional Cardiology? It might be defined as a branch of cardiology which focuses specifically on the treatment and management of structural heart diseases in catheter-based laboratories. The current status, new updates, and future directions related to Interventional cardiology have recently been published.^[1–4]^ Interventional procedures are becoming increasingly common and they are now becoming the preferred modes of treatment among patients with specific cardiac disorders. Percutaneous coronary intervention (PCI), which is often associated with earlier hospital discharge,^[5]^ is 1 among the most common interventional procedures which are carried out in PCI-capable centers. Management of acute coronary syndrome (ACS) including ST-segment elevation myocardial infarction (STEMI) and non-STEMI,^[6]^ and also several types of nonsevere multivessel coronary artery diseases (MVCADs),^[7]^ and unprotected left main coronary artery diseases (ULMCAD) is nowadays possible with PCI.^[8]^ Even though PCI might be an acceptable choice in most of the patients, certain patients’ conditions and the extent of coronary lesions might restrict its use, thereby shifting its place to coronary artery bypass surgery (CABG).^[9]^

However, the question which has to be raised at this particular point concerns the identification of patients who might benefit from PCI. Recently, the Synergy Between PCI With Taxus and CABG (SYNTAX) score was developed.^[10]^ It is a tool which takes into consideration the anatomical features of the coronary lesions as a guide to assess patients who will be eligible for PCI.^[11]^

Nevertheless, should the SYNTAX score be integrated in Interventional Cardiology? Should it really be considered as a vital decision-making tool in PCI? To confirm the importance of this score, we aimed to systematically compare the postinterventional adverse cardiovascular outcomes which were observed in patients who were allotted a low versus a high SYNTAX score.

## 2 Methods

### 2.1 Searched databases and strategies

Following the PRISMA guideline,^[12]^ randomized controlled trials and observational studies published from January 2007 to January 2017 were identified through MEDLINE, EMBASE, and the Cochrane databases using the searched terms or keywords which were listed below:

1. SYNTAX score
2. SYNTAX score and percutaneous coronary intervention
3. SYNTAX score and interventional cardiology
4. SYNTAX score and coronary angioplasty
5. SYNTAX score and PCI
6. SYNTAX score and coronary artery disease (CAD)
7. SYNTAX score and coronary stenting

It should be noted that the reference lists of suitable publications were also checked for relevant articles.

Our searched criteria were limited to English publications involving humans only.

### 2.2 Inclusion criteria

Studies were included if they met the following criteria:

1. They were randomized trials or observational cohorts comparing PCI in patients who were allotted a low versus a high SYNTAX score.
2. They reported adverse clinical outcomes as their major endpoints.
3. They included any type of participants with CAD.

### 2.3 Exclusion criteria

Studies were excluded based on the following criteria:

1. They were meta-analysis, case-control studies, or letters to editors.
2. They compared only CABG in patients who were allotted a low versus a high SYNTAX score.
3. They did not report adverse clinical outcomes as their major endpoints.
4. They were duplicated studies or they were different studies which involved the same trial.

### 2.4 Types of participants, outcomes, and follow-ups

This research article included several types of patients with CAD who were revascularized by PCI. The different types of participants (Table 1) were patients with any type of CAD; ST-segment elevation MI (STEMI); non-ST-segment elevation MI (NSTEMI); left main CAD (LMCAD); MVCAD; and three-vessel CAD.

**Table 1:**
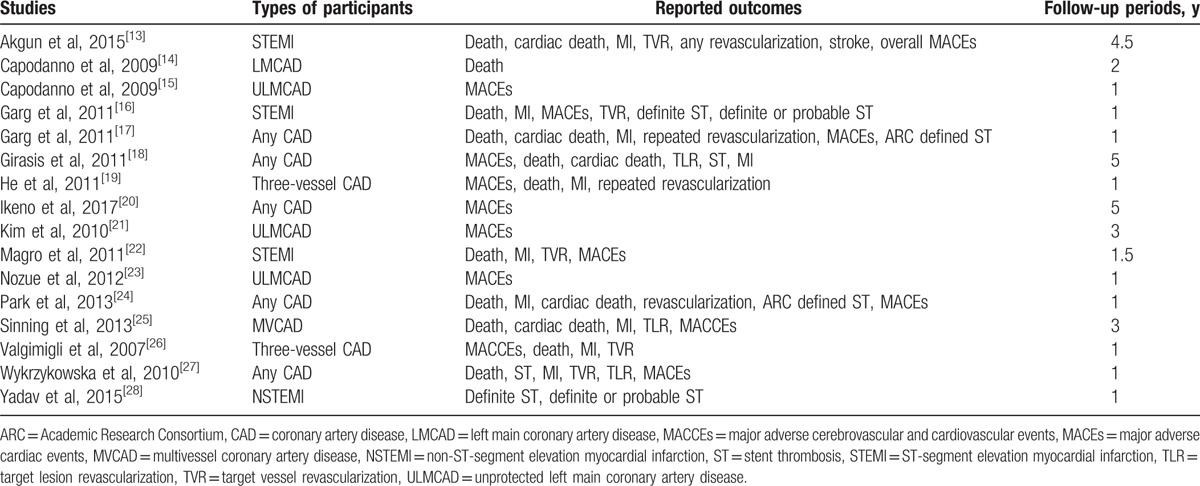
Type of participants, reported outcomes, and follow-ups.

The outcomes which were assessed included the following:

1. All-cause mortality.
2. Cardiac death.
3. Myocardial infarction (MI).
4. Major adverse cardiac events (MACEs), which were defined as the combination of death, MI, and revascularization. Major adverse cerebrovascular and cardiovascular events (MACCEs), which consisted of death, MI, stroke, and revascularization, were also included in the same category as MACEs and analyzed together.
5. Repeated revascularization which consisted of target vessel revascularization (TVR) and/or target lesion revascularization (TLR).
6. Stent thrombosis (ST), which was defined according to the Academic Research Consortium (ARC)^[29]^ and which was composed of definite and probable ST.

The follow-up periods varied from study to study. Most of the studies had a follow-up period of 1 year, as shown in Table 1.

### 2.5 Definitions

The SYNTAX score was classified into 3 different categories known as tertiles, as given below:

1. Tertile I was defined as patients with the lowest SYNTAX score.
2. Tertile II was defined as patients with an intermediate/mid SYNTAX score.
3. Tertile III was defined as patients with the highest SYNTAX score.

This information has been represented in Table 2.

**Table 2:**
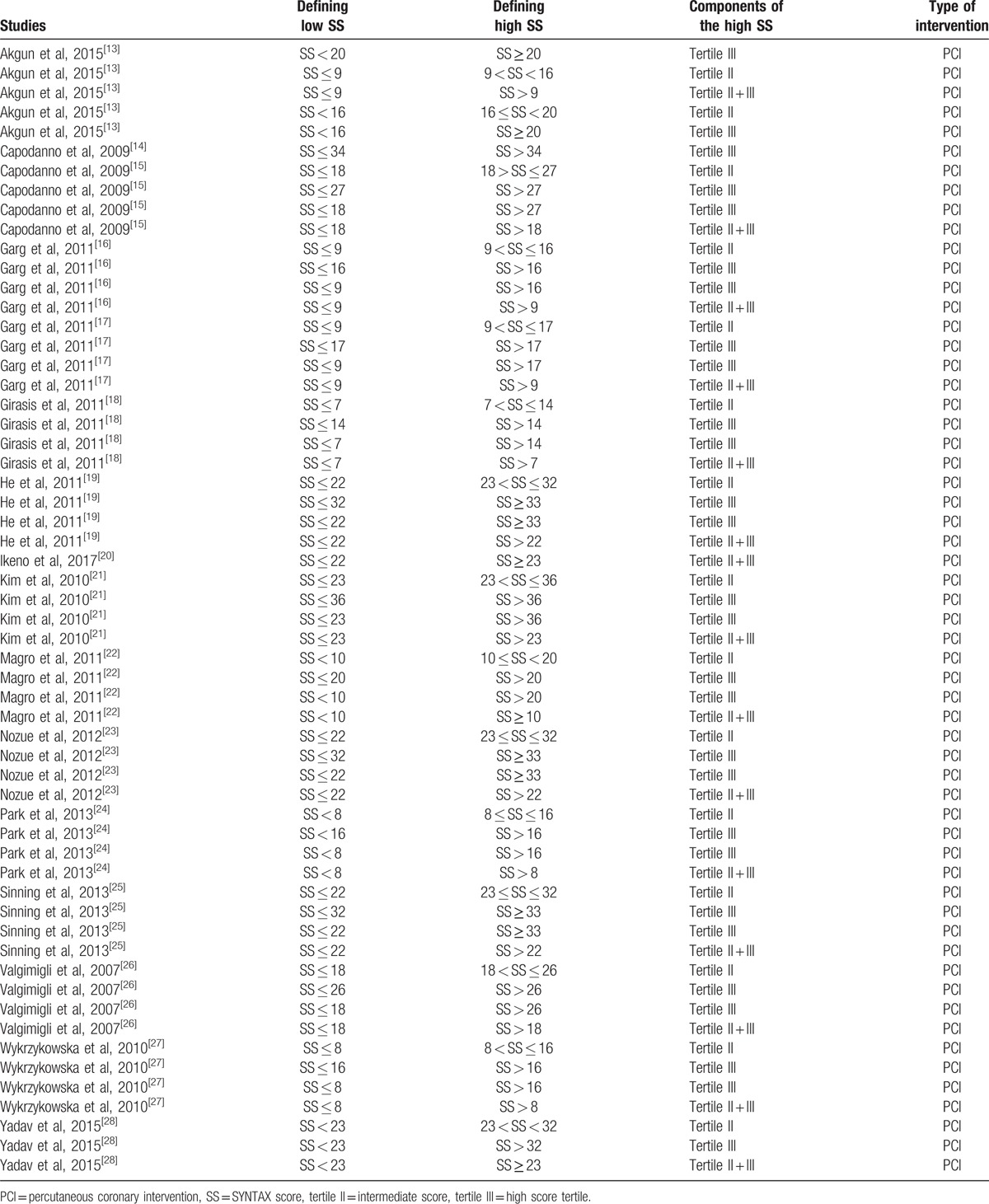
Definitions of low versus high SYNTAX score (the different tertiles).

### 2.6 Data extraction and quality assessment

Studies which were considered eligible for this analysis were first of all carefully assessed by 3 independent reviewers (P.K.B., Y.S., and A.B.) to ensure that they satisfied the eligibility criteria of this research article.

The following data were extracted by the same 3 reviewers:

1. Names of the first author
2. Year of publication
3. Types of study which were reported
4. Periods of participants’ enrollment
5. Types of participants which were included
6. Baseline characteristics of the participants (including the mean age, percentage of male participants, percentage of participants suffering from comorbidities such as hypertension, dyslipidemia, and diabetes mellitus)
7. Total number of participants who were allotted a low SYNTAX score
8. Total number of participants who were allotted a higher SYNTAX score
9. The different tertiles (tertiles I, II, and III)
10. The clinical outcomes and the number of events which were reported within the study and the control groups, respectively
11. The follow-up periods
12. The interventional procedures which were followed
13. Details about the quality of the trials and observational studies

Quality assessment was carried out separately for the trials and the observational cohorts using the Cochrane Handbook^[30]^ and the Newcastle Ottawa Scale (NOS),^[31]^ respectively. The trials were assessed for the 6 components which were recommended by the Cochrane Collaborations, whereby scores were given in accordance to a low, unclear and high risk of bias, and the total score which was obtained by each trial was graded from A to E, whereby A implied a very low risk of bias, B and C implied low to moderate risk of bias, and E indicated a very high risk of bias.

For the observational studies, a star system assessment was carried out whereby stars were allotted based on certain components which were required during quality assessment. A maximum total number of 9 stars were possible which implied a very low risk of bias.

Any disagreement which followed whether during the data extraction process or the quality assessment was discussed among the reviewers. However, if a consensus could not be reached, a decision was finalized by the fourth reviewer (F.H.).

### 2.7 Statistical analysis

This is a meta-analysis of several studies, including different types of patients who underwent revascularization by PCI. Therefore, inconsistency across the studies was possible. To obtain a more consistent result, heterogeneity^[32]^ across the studies was calculated/evaluated/assessed using the *Q* statistic test (*P* ≤ .05 was considered statistically significant) and the *I*^2^ statistic test (high percentage = higher heterogeneity [whereby a random-effects model was used if a value greater than 50% was obtained] and low percentage = lower heterogeneity [whereby a fixed-effects model was used if a value equal to or less than 50% was obtained]).

The analysis was carried out whereby risk ratios (RRs) with 95% confidence intervals (CIs) were calculated by the RevMan version 5.3 software.

Sensitivity analysis was also carried out by excluding each study one by one and observing any significant difference in subgroup analysis in comparison to the main results.

In addition, publication bias,^[32]^ which was also possible across the studies, was visually estimated by assessing graphical plots through RevMan 5.3.

### 2.8 Ethical approval

Ethical or board review approval and patients’ consents were not required for meta-analyses.

## 3 Results

### 3.1 Searched outcomes

A careful search through the electronic databases which was carried out by those 3 reviewers resulted in a total number of 1147 articles as listed below:

1. MEDLINE: 401 articles
2. EMBASE: 423 articles
3. Cochrane database: 234 articles
4. Reference lists of relevant articles: 89 articles

The 3 reviewers carefully assessed the titles and abstracts. Based on this assessment, 1004 articles were eliminated since they were not considered relevant to the scope of this research.

In all, 143 full-text articles were assessed for eligibility. Further articles were eliminated due to the following reasons:

1. They were meta-analysis, case-control studies, and letters to editors (n = 4).
2. They only compared adverse outcomes in patients who were revascularized by PCI with a low SYNTAX score versus CABG with a high SYNTAX score (n = 12).
3. They only compared CABG patients who were allotted a low versus a high SYNTAX score (n = 8).
4. They were duplicated studies or they were different studies which were associated with similar trials (n = 103).

**Figure 1:**
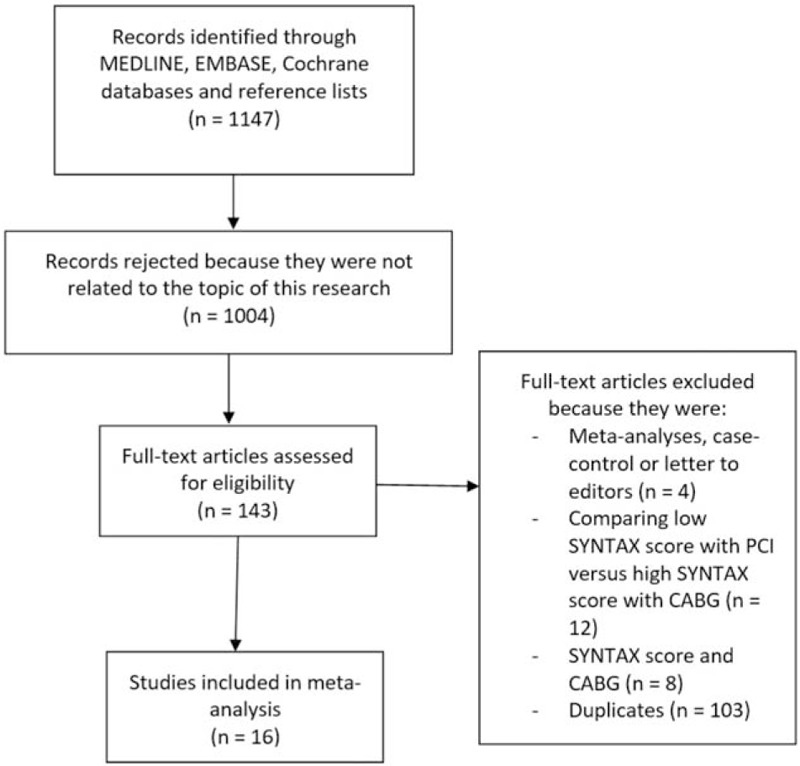
Flow diagram which represents the study selection.

Finally, only 16 full-text articles^[13–28]^ (6 randomized trials and 10 observational studies) were selected for this analysis as shown in Fig. 1.

### 3.2 General features of the studies

Six randomized trials and 10 observational cohorts with a total number of 19,751 participants (8589 participants with a low SYNTAX score versus 11,162 participants with a high SYNTAX score) were included in the main analysis. Patients’ enrollment periods varied from the years 2000 to 2010 as shown in Table 3.

**Table 3:**
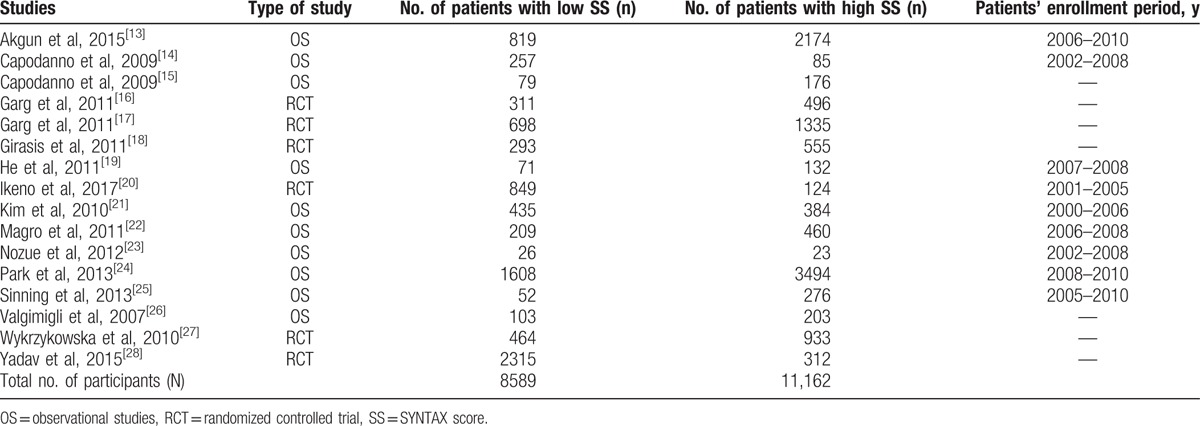
General features of the studies which were included.

After the quality assessment, a grade B was allotted to the trials, whereas number of stars allotted to the observational studies varied from 6 to 8 stars.

### 3.3 Baseline features of the participants

The baseline characteristics of the participants were summarized in Table 4. A mean age ranging from 50.0 to 71.0 years was noted among the participants. Most of the studies reported a majority of male compared with female participants as shown in Table 4. The percentage of participants with hypertension, dyslipidemia, current smoking, and those who suffered from type 2 diabetes mellitus were also listed in Table 4. According to the baseline features, almost no significant differences were observed among participants within the low SYNTAX and high SYNTAX groups, with the exception of a few studies.

**Table 4:**
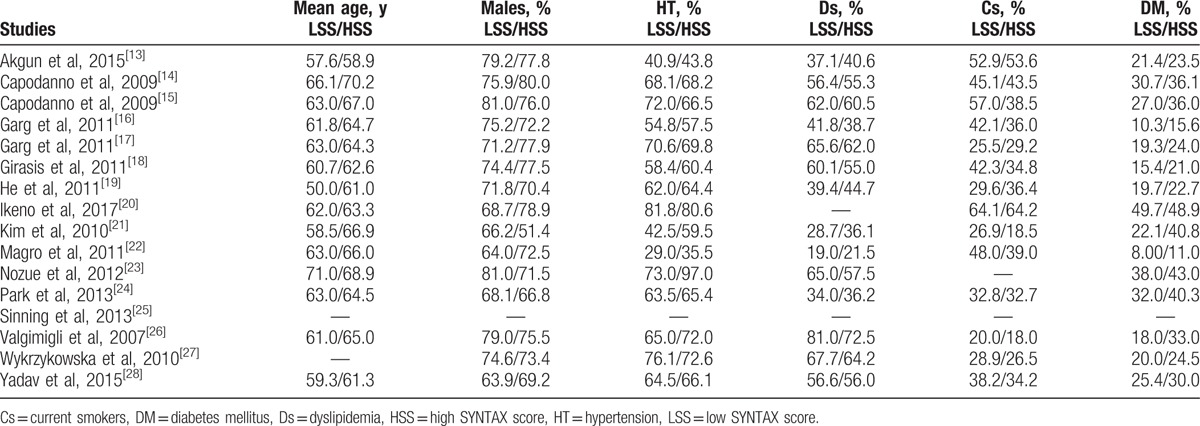
Baseline features (for the participants with a low vs a higher SYNTAX score).

### 3.4 Main results of this analysis

The results were subdivided into different categories, as described in the following subsections.

#### 3.4.1 Low SYNTAX score versus higher SYNTAX score (tertile II + III)

First of all, after PCI, adverse cardiovascular outcomes associated with a low SYNTAX score was compared with adverse outcomes associated with a higher SYNTAX score (tertile II + III).

**Figure 2:**
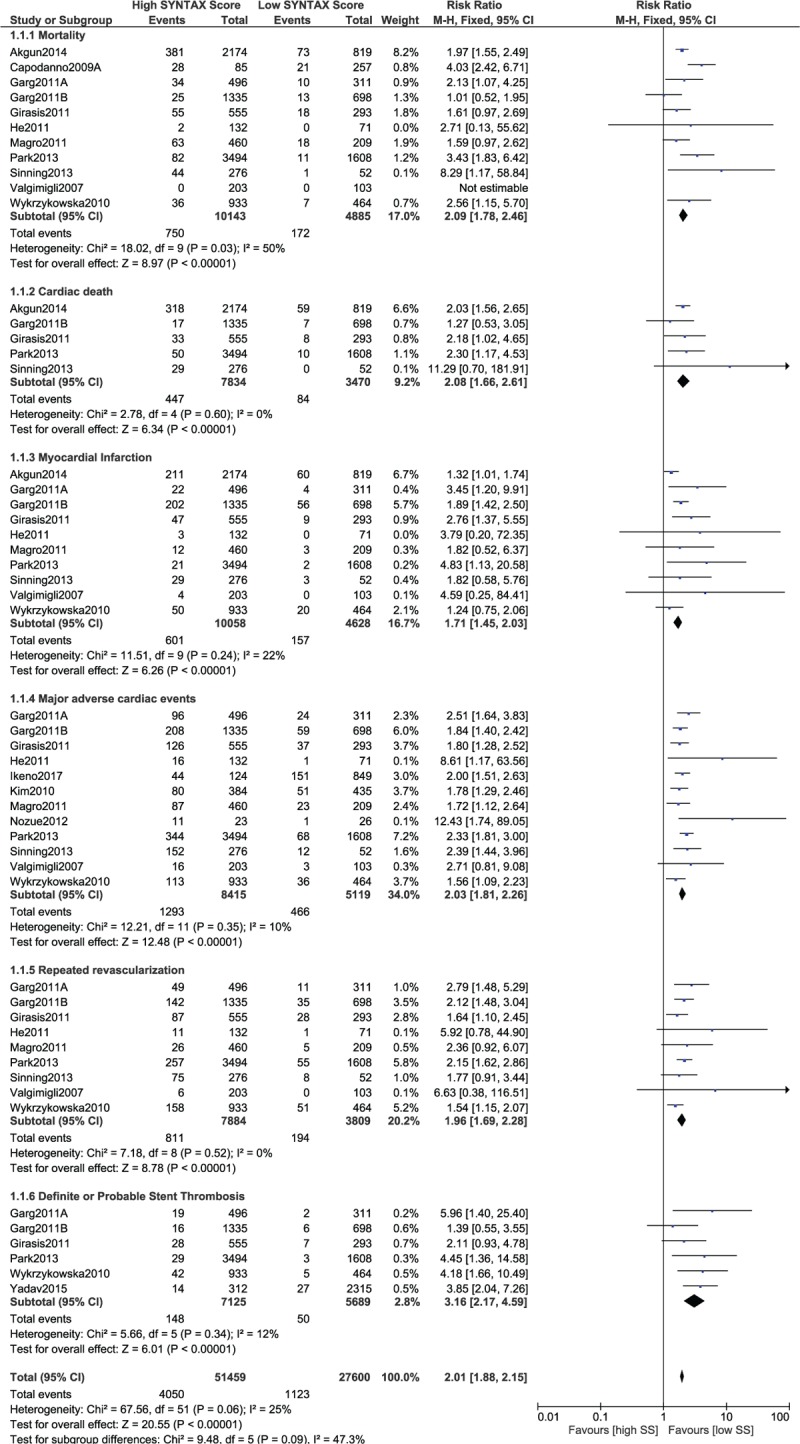
Postinterventional adverse cardiovascular outcomes which were observed between a low versus a higher (tertiles II and III) SYNTAX score.

The current results showed mortality to be significantly higher with the higher SYNTAX score (RR 2.09, 95% CI 1.78–2.46, *P* = .00001, as shown in Fig. 2). Cardiac death also significantly favored a low SYNTAX score (RR 2.08, 95% CI 1.66–2.61, *P* = .00001). Similarly, MI, MACEs, repeated revascularization, and stent thrombosis were significantly higher with a high SYNTAX score (RR 1.71, 95% CI 1.45–2.03, *P* = .00001; RR 2.03, 95% CI 1.81–2.26, *P* = .00001; RR 1.96, 95% CI 1.69–2.28, *P* = .00001; and RR 3.16, 95% CI 2.17–4.59, *P* = .00001, respectively, as shown in Fig. 2).

**Figure 3:**
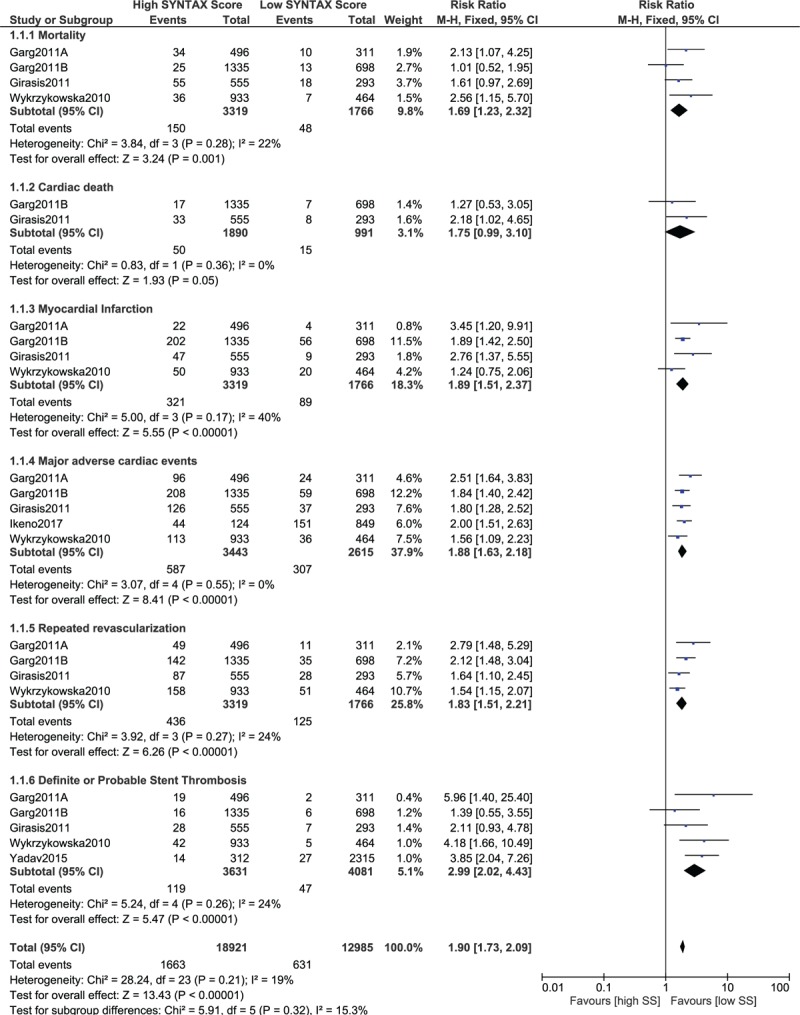
Postinterventional adverse cardiovascular outcomes which were observed between a low versus a higher (tertiles II and III) SYNTAX score using data which were obtained only from randomized controlled trials.

It should be noted that while carrying out this analysis, data which were obtained from observational studies were combined with data which were obtained from randomized controlled trials. Therefore, another analysis was separately carried out involving only data which were obtained from randomized trials to observe any change in the results. However, similar to the previous results, this separate analysis also showed that significantly higher mortality, cardiac death, MI, MACEs, repeated revascularization, and stent thrombosis were observed with a high SYNTAX score (RR 1.69, 95% CI 1.23–2.32, *P* = .001; RR 1.75, 95% CI 0.99–3.10, *P* = .05; RR 1.89, 95% CI 1.51–2.37, *P* = .00001; RR 1.88, 95% CI 1.63–2.18, *P* = .00001; RR 1.83, 95% CI 1.51–2.21, *P* = .00001; and RR 2.99, 95% CI 2.02–4.43, *P* = .00001, respectively, as shown in Fig. 3).

#### 3.4.2 Low SYNTAX score versus higher SYNTAX score (tertile II + III) with specific limits/ranges of score

The score range was completely omitted in the above-shown analysis. A low SYNTAX score with any range was compared with the corresponding higher score. However, the analysis was further divided into several subsets with different score limits.

**Figure 4:**
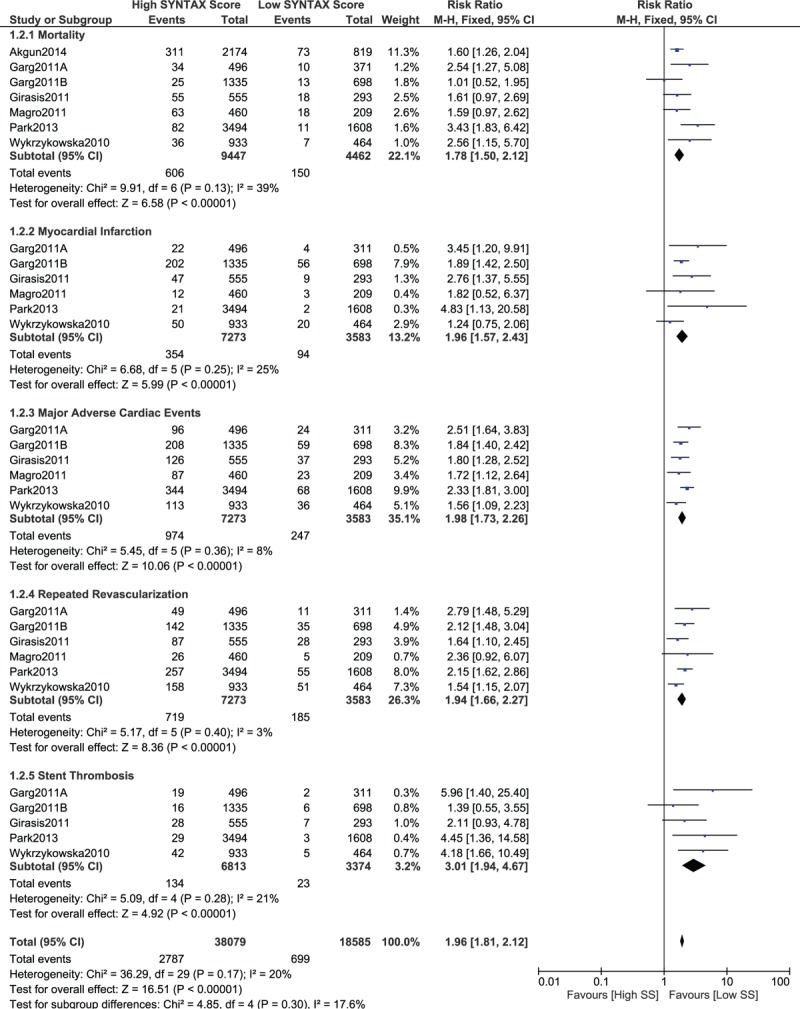
Postinterventional adverse cardiovascular outcomes which were observed between a low (SS ≤ 10) versus a higher (tertiles II and III) SYNTAX score. SS = SYNTAX score.

When the adverse outcomes were compared in patients who were allotted a low SYNTAX score of ≤10 versus a higher score, significantly higher mortality, MI, MACEs, repeated revascularization, and stent thrombosis were still associated with the higher score (RR 1.78, 95% CI 1.50–2.12, *P* = .00001; RR 1.96, 95% CI 1.57–2.43, *P* = .00001; RR 1.98, 95% CI 1.73–2.26, *P* = .00001; RR 1.94, 95% CI 1.66–2.27, *P* = .00001; and RR 3.01, 95% CI 1.94–4.67, *P* = .00001, respectively, as shown in Fig. 4).

**Figure 5:**
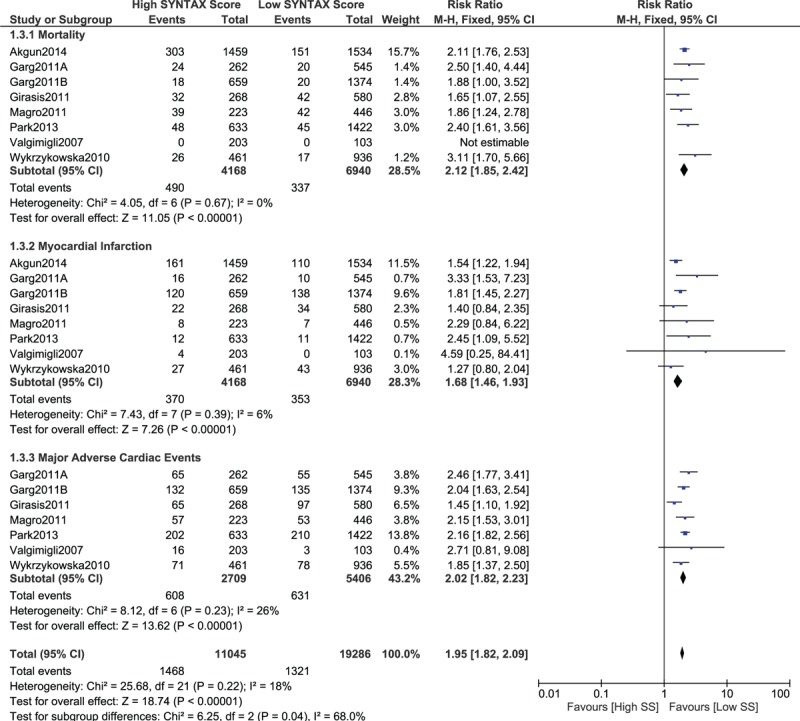
Postinterventional adverse cardiovascular outcomes which were observed between a low (10 > SS ≤ 20) versus a higher (tertiles II and III) SYNTAX score. SS = SYNTAX score.

**Figure 6:**
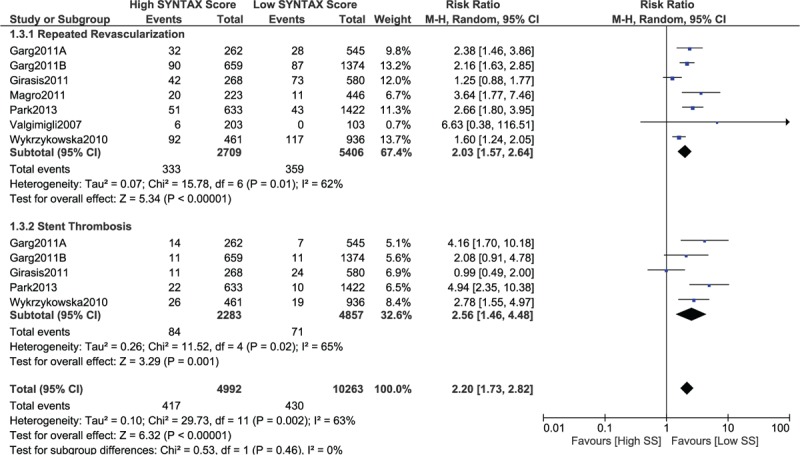
Postinterventional adverse cardiovascular outcomes which were observed between a low (10 > SS ≤ 20) versus a higher (tertiles II and III) SYNTAX score. SS = SYNTAX score.

When a lower SYNTAX score 10 > SYNTAX score ≤ 20 was considered as the lower score range, mortality, MI and MACEs still significantly favored the lower score (RR 2.12, 95% CI 1.85–2.42, *P* = .00001; RR 1.68, 95% CI 1.46–1.93, *P* = .00001; and RR 2.02, 95% CI 1.82–2.23, *P* = .00001, respectively, as shown in Fig. 5). In addition, repeated revascularization and stent thrombosis were also significantly in favor of a lower SYNTAX score (RR 2.03, 95% CI 1.57–2.64, *P* = .00001 and RR 2.56, 95% CI 1.46–4.48, *P* = .001, respectively, as shown in Fig. 6).

**Figure 7:**
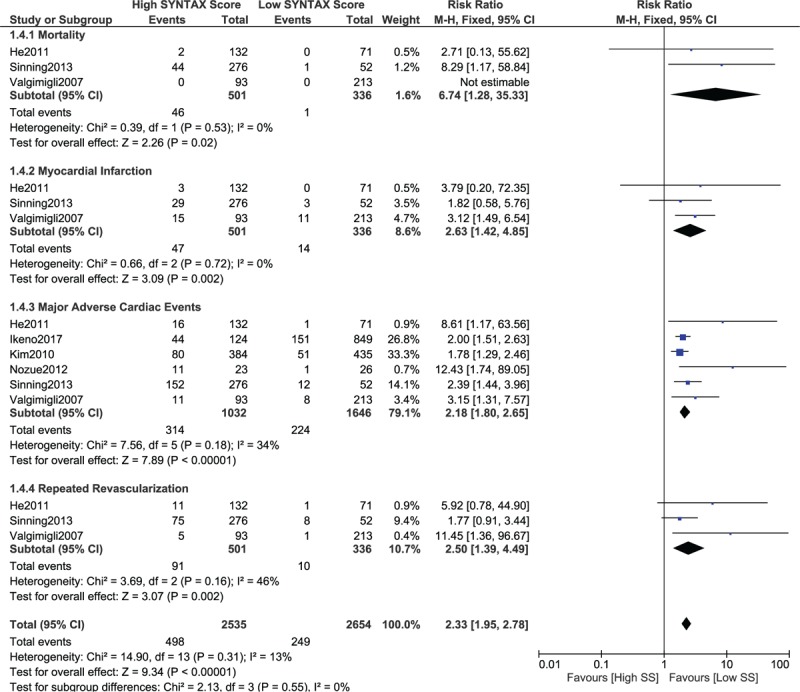
Postinterventional adverse cardiovascular outcomes which were observed between a low (20 > SS < 30) versus a higher (tertiles II and III) SYNTAX score. SS = SYNTAX score.

When a score range 20 > SYNTAX score < 30 was considered for a low SYNTAX score, mortality, MI, MACEs, and repeated revascularization were still significantly higher (RR 6.74, 95% CI 1.28–35.33, *P* = .02; RR 2.63, 95% CI 1.42–4.85, *P* = .002; RR 2.18, 95% CI 1.80–2.65, *P* = .00001; and RR 2.50, 95% CI 1.39–4.49, *P* = .002, respectively, as shown in Fig. 7).

**Figure 8:**
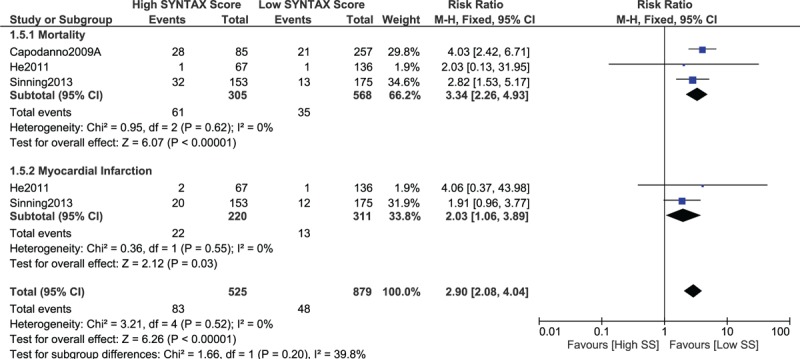
Postinterventional adverse cardiovascular outcomes which were observed between a low (30 > SS < 40) versus a higher (tertiles II and III) SYNTAX score. SS = SYNTAX score.

**Figure 9:**
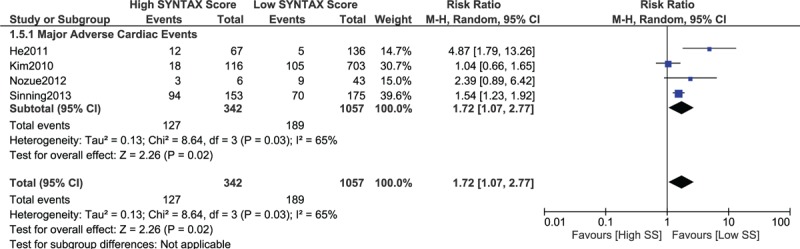
Postinterventional adverse cardiovascular outcomes which were observed between a low (30 > SS < 40) versus a higher (tertiles II and III) SYNTAX score. SS = SYNTAX score.

When a score range 30 > SYNTAX score < 40 was considered in the lower SYNTAX range, mortality and MI were still significantly higher with a high SYNTAX score (RR 3.34, 95% CI 2.26–4.93, *P* = .00001 and RR 2.03, 95% CI 1.06–3.89, *P* = .03, respectively; Fig. 8). In addition, MACEs were also significantly higher with a high SYNTAX score (RR 1.72, 95% CI 1.07–2.77, *P* = .02; Fig. 9).

#### 3.4.3 Low versus intermediate SYNTAX score (tertile I vs tertile II)

**Figure 10:**
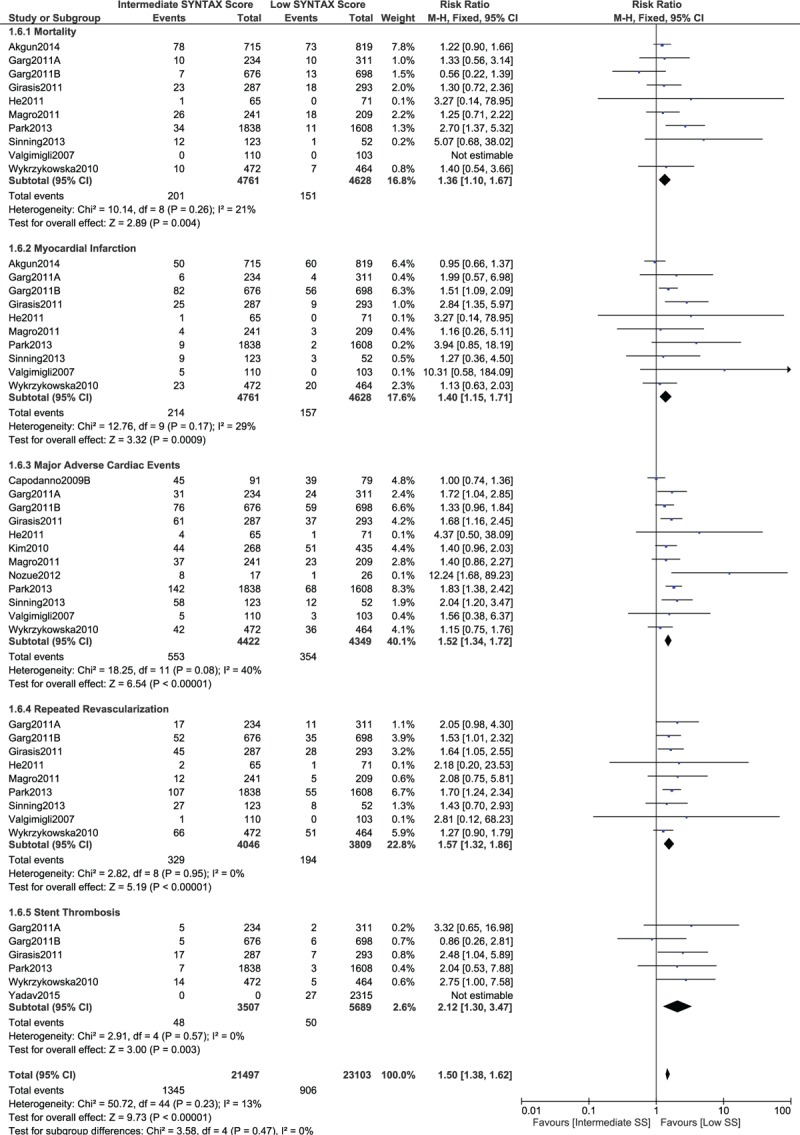
Postinterventional adverse cardiovascular outcomes which were observed between a low versus an intermediate (tertile II) SYNTAX score.

When a low SYNTAX score was compared with an intermediate SYNTAX score, mortality, MI, MACEs, repeated revascularization, and stent thrombosis were still significantly lower with a lower SYNTAX score (RR 1.36, 95% CI 1.10–1.67, *P* = .004; RR 1.40, 95% CI 1.15–1.71, *P* = .0009; RR 1.52, 95% CI 1.34–1.72, *P* = .00001; RR 1.57, 95% CI 1.32–1.86, *P* = .00001; and RR 2.12, 95% CI 1.30–3.47, *P* = .003, respectively, as shown in Fig. 10).

#### 3.4.4 Low versus high SYNTAX score (tertile I vs tertile III)

**Figure 11:**
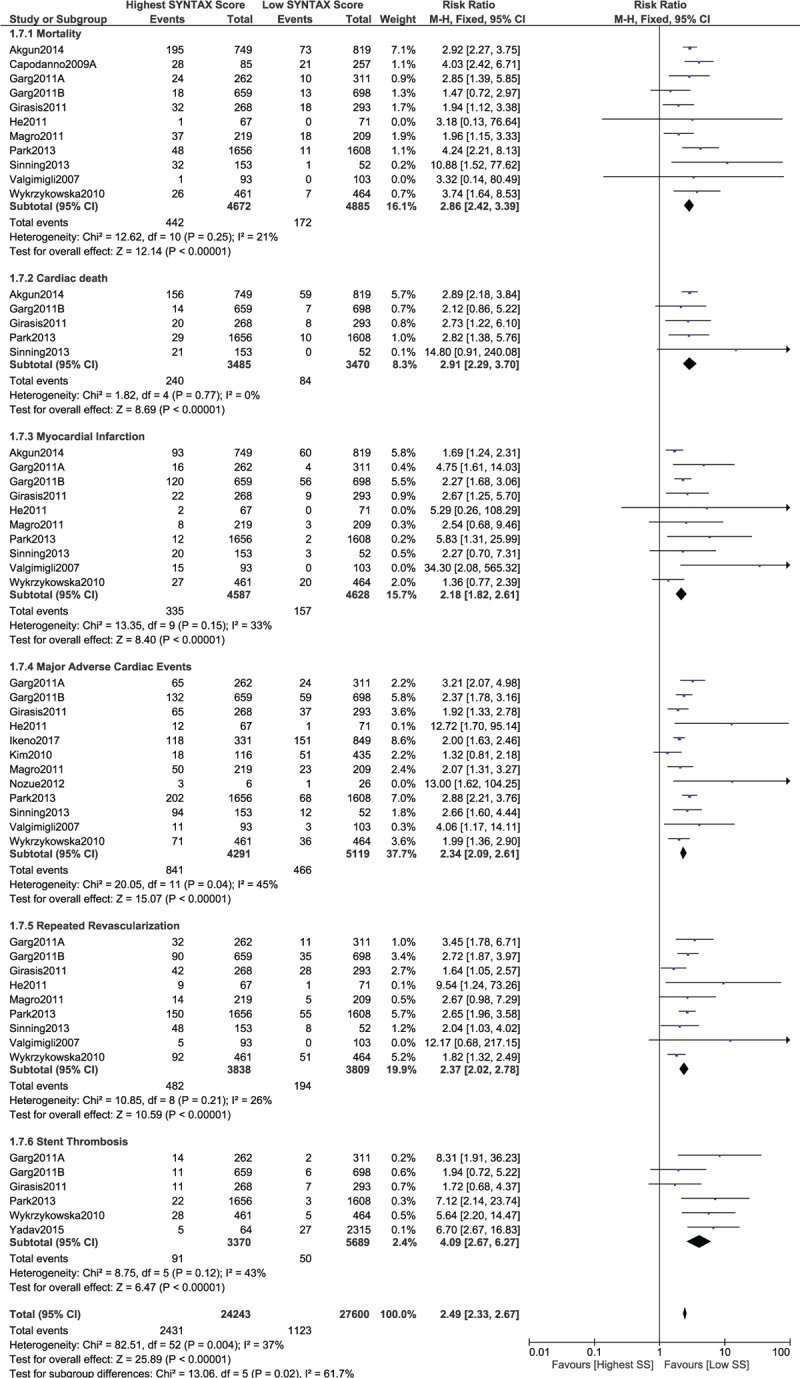
Postinterventional adverse cardiovascular outcomes which were observed between a low versus a high (tertile III) SYNTAX score.

When a low SYNTAX score was compared with a high SYNTAX score, mortality, cardiac death, MI, MACEs, repeated revascularization, and stent thrombosis significantly favored a lower score (RR 2.86, 95% CI 2.42–3.39, *P* = .00001; RR 2.91, 95% CI 2.29–3.70, *P* = .00001; RR 2.18, 95% CI 1.82–2.61, *P* = .00001; RR 2.34, 95% CI 2.09–2.61, *P* = .00001; RR 2.37, 95% CI 2.02–2.78, *P* = .00001; and RR 4.09, 95% CI 2.67–6.27, *P* = .00001, respectively, as shown in Fig. 11).

#### 3.4.5 Low versus higher SYNTAX score (tertile II + III) in a subset of patients with STEMI

**Figure 12:**
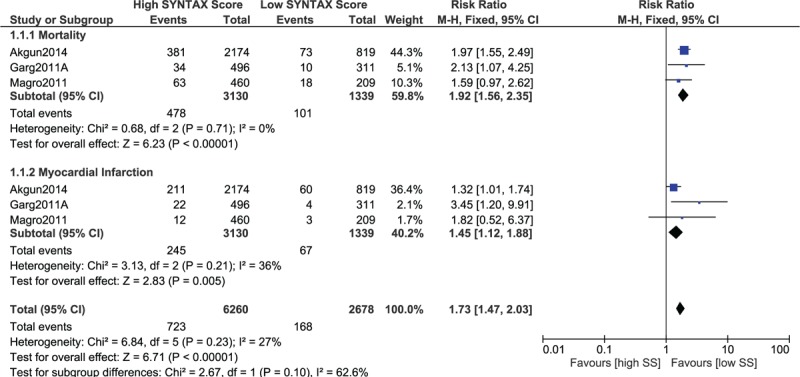
Postinterventional adverse cardiovascular outcomes which were observed between a low versus a higher (tertile II + III) SYNTAX score in patients with STEMI. STEMI = ST-segment elevation myocardial infarction.

**Figure 13:**
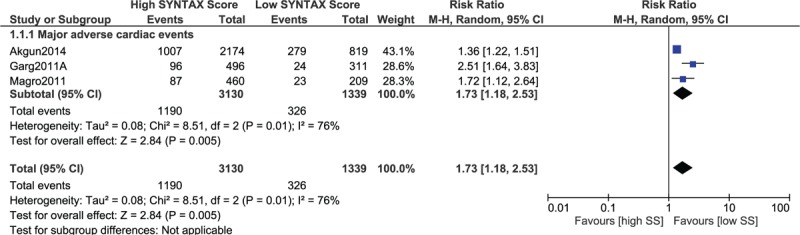
Postinterventional major adverse cardiac events which were observed between a low versus a higher (tertile II + III) SYNTAX score in patients with STEMI. STEMI = ST-segment elevation myocardial infarction.

A separate analysis was carried out involving only patients with STEMI. The results were still in favor of a low SYNTAX score, whereby mortality and MI were significantly lower in STEMI patients with a low SYNTAX score (RR 1.92, 95% CI 1.56–2.35, *P* = .00001 and RR 1.45, 95% CI 1.12–1.88, *P* = .005, respectively; Fig. 12). In addition, MACEs also significantly favored a low SYNTAX score in these patients with STEMI (RR 1.73, 95% CI 1.18–2.53, *P* = .005; Fig. 13).

### 3.5 Publication bias

**Figure 14:**
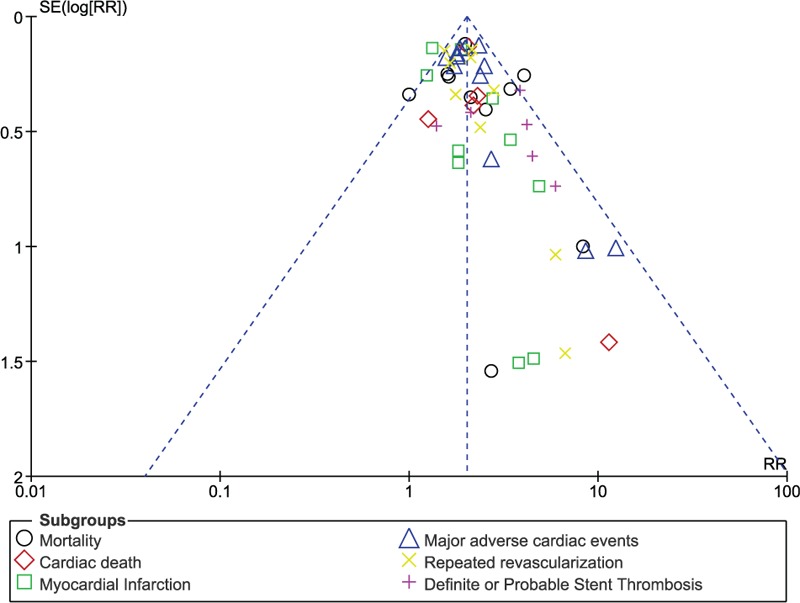
Funnel plot (A) representing publication bias.

**Figure 15:**
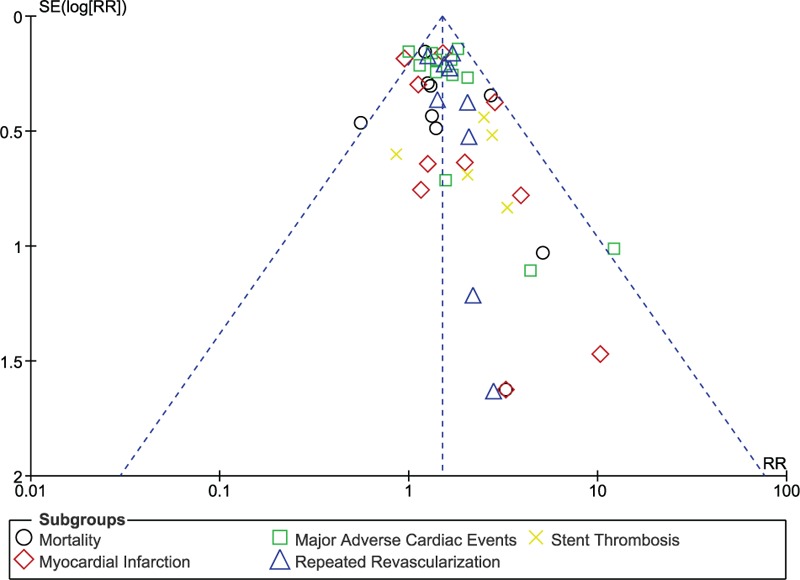
Funnel plot (B) representing publication bias.

Sensitivity analysis did not show any deviation from these main results. Moreover, based on a visual evaluation of the funnel plots, there has been very little evidence for the existence of publication bias across all the eligible studies which were involved in assessing the relevant cardiovascular outcomes (Figs. 14 and 15).

## 4 Discussion

Even if the SYNTAX score is not among the newest angiographic tools which have been used in clinical practice, it was the most common one to be used to stratify patients who would benefit from either PCI or CABG until recently, newer scientific reports showed its application in Interventional cardiology, whereby it could potentially stratify those patients who would most probably benefit from PCI alone.

In this analysis, we demonstrated the potential benefits of the SYNTAX score and its potential application in Interventional cardiology. These current results showed that when a low SYNTAX score was compared with an intermediate or higher SYNTAX score, significantly lower adverse cardiovascular outcomes were associated with the lower score. A consistent result was obtained among all the subgroups. This analysis included patients with STEMI, NSTEMI, ULMCAD, and MVCAD. However, even when patients with STEMI were separately analyzed, a low SYNTAX score was still significantly associated with lower adverse outcomes.

A subanalysis of the shinshu prospective multicenter study of elderly patients with coronary artery disease undergoing percutaneous coronary intervention registry also supported the results of this current analysis showing that a lower SYNTAX score predicted a lower incidence of MACEs.^[33]^ The authors also stated that the SYNTAX score should be considered an important parameter to improve risk stratification in similar patients. Even if the study satisfied most of the eligibility criteria for this analysis, it was not included among the eligible studies because the patients also suffered from heart failure.

The gene polymorphism, platelet reactivity, and the syntax score study,^[34]^ which was a prospective, multicentered cohort including 1053 patients with NSTEMI who underwent coronary revascularization by PCI, and who were treated with clopidogrel after this invasive procedure, showed higher platelet reactivity to be independently associated with an increased risk of MACEs only in patients with a high SYNTAX score. This association was not visible in patients with lower SYNTAX scores.

In addition, a recently published meta-analysis also showed a positive aspect of the SYNTAX score in predicting all-cause mortality in patients who were treated by PCI, indicating its importance in Interventional cardiology.^[35]^ However, in this same analysis, the authors stated that the SYNTAX score often overestimated the risk of MACEs. However, in this current analysis, MACEs, which are among the vital clinical endpoints in Interventional cardiology,^[36]^ were not overestimated.

Nevertheless, it should be noted that this current analysis has almost all the features that are required to be considered a well-carried out meta-analysis in terms of the total number of studies and participants, low bias risks across the studies, low levels of heterogeneity in almost all the subgroups, and well-presented robust results. Therefore, the SYNTAX score should be expected to at least be integrated in Interventional cardiology, despite emerging newer clinical tools,^[37–39]^ which should but might take longer to find a place in Interventional cardiology.

### 4.1 Novelty

New features in this analysis include the following:

1. A new idea in Interventional cardiology.
2. An important potential tool has been studied.
3. This meta-analysis might be among the first analyses demonstrating the use of this new tool in Interventional cardiology.
4. A large number of participants who underwent revascularization by PCI were included.
5. Low SYNTAX score was compared with higher (tertiles II and III) SYNTAX score.
6. Low SYNTAX score was compared with intermediate (tertile II) SYNTAX score.
7. Low SYNTAX score was compared with high (tertile III) SYNTAX score.
8. Different range limits of SYNTAX score were also compared.
9. Randomized trials were also separately analyzed.
10. Several adverse cardiovascular outcomes were analyzed.
11. Patients who suffered from STEMI were also separately analyzed to show a result specifically for this particular subgroup of patients.

### 4.2 Limitations

The limitations of this study were as follows:

1. Different studies reported different follow-up periods which might have influenced the result. However, most of the studies reported a follow-up period of 1 year only.
2. Several types of patients with CAD were analyzed together. However, when patients with STEMI were separately analyzed, the same results were obtained.
3. Data obtained from observational studies and randomized trials were combined and analyzed. However, even when randomized trials were separately analyzed, a similar result was obtained, partly solving this limitation.
4. The range limit of the scores was not exactly the same; small variations might have been responsible for the moderate level of heterogeneity observed in certain subgroups.

## 5 Conclusions

This analysis is a confirmatory piece of evidence to show that the application of the SYNTAX score in Interventional cardiology is apparently relevant. A low SYNTAX score was associated with significantly better cardiovascular outcomes in comparison with a higher SYNTAX score. Therefore, the SYNTAX score is an angiographic tool which might possibly be of some importance and should be applied in clinical practice.
